# Alternative splicing of *U2AF1* reveals a shared repression mechanism for duplicated exons

**DOI:** 10.1093/nar/gkw733

**Published:** 2016-08-26

**Authors:** Jana Kralovicova, Igor Vorechovsky

**Affiliations:** University of Southampton, Faculty of Medicine, Southampton SO16 6YD, UK

## Abstract

The auxiliary factor of U2 small nuclear ribonucleoprotein (U2AF) facilitates branch point (BP) recognition and formation of lariat introns. The gene for the 35-kD subunit of U2AF gives rise to two protein isoforms (termed U2AF35a and U2AF35b) that are encoded by alternatively spliced exons 3 and Ab, respectively. The splicing recognition sequences of exon 3 are less favorable than exon Ab, yet U2AF35a expression is higher than U2AF35b across tissues. We show that U2AF35b repression is facilitated by weak, closely spaced BPs next to a long polypyrimidine tract of exon Ab. Each BP lacked canonical uridines at position -2 relative to the BP adenines, with efficient U2 base-pairing interactions predicted only for shifted registers reminiscent of programmed ribosomal frameshifting. The BP cluster was compensated by interactions involving unpaired cytosines in an upstream, EvoFold-predicted stem loop (termed ESL) that binds FUBP1/2. Exon Ab inclusion correlated with predicted free energies of mutant ESLs, suggesting that the ESL operates as a conserved rheostat between long inverted repeats upstream of each exon. The isoform-specific U2AF35 expression was U2AF65-dependent, required interactions between the U2AF-homology motif (UHM) and the α6 helix of U2AF35, and was fine-tuned by exon Ab/3 variants. Finally, we identify tandem homologous exons regulated by U2AF and show that their preferential responses to U2AF65-related proteins and SRSF3 are associated with unpaired pre-mRNA segments upstream of U2AF-repressed 3′ss. These results provide new insights into tissue-specific subfunctionalization of duplicated exons in vertebrate evolution and expand the repertoire of exon repression mechanisms that control alternative splicing.

## INTRODUCTION

U2AF is a stable heterodimer that facilitates recruitment of the U2 small nuclear ribonucleoprotein (snRNP) to the branch point (BP) (1–3). It consists of a 65-kD subunit (U2AF65), which interacts with conserved, Y-rich sequences upstream of 3′ splice sites (3′ss) known as polypyrimidine tracts (PPTs) (2), and a 35-kD subunit (U2AF35), which contacts almost invariant AG dinucleotides at 3′ss and stabilizes U2AF65 binding (4–6). Each U2AF subunit is essential for viability (7–10). Recent global transcriptomic studies showed that the knockdown of human subunits affected preferentially alternative RNA splicing and polyadenylation without widespread failure to recognize 3′ss of constitutive exons (11,12), consistent with U2AF binding to a subset of 3′ss (11,13) and with its role in transcription and gene termination (14–17). Depletion of each subunit altered usage of U2AF-dependent exons almost exclusively in the same direction (11,12), in agreement with their parallel requirements for 3′ss recognition in yeast and their functional collaboration *in vivo* (10). U2AF35 can self-interact (18) and knockdown of U2AF35 or overexpression of U2AF65 activated an identical cryptic 3′ss (19), suggesting that stoichiometry of the two subunits is important for accurate 3′ss selection, but regulatory networks that maintain their equilibrium in the cell are poorly understood.

**Figure 1.**
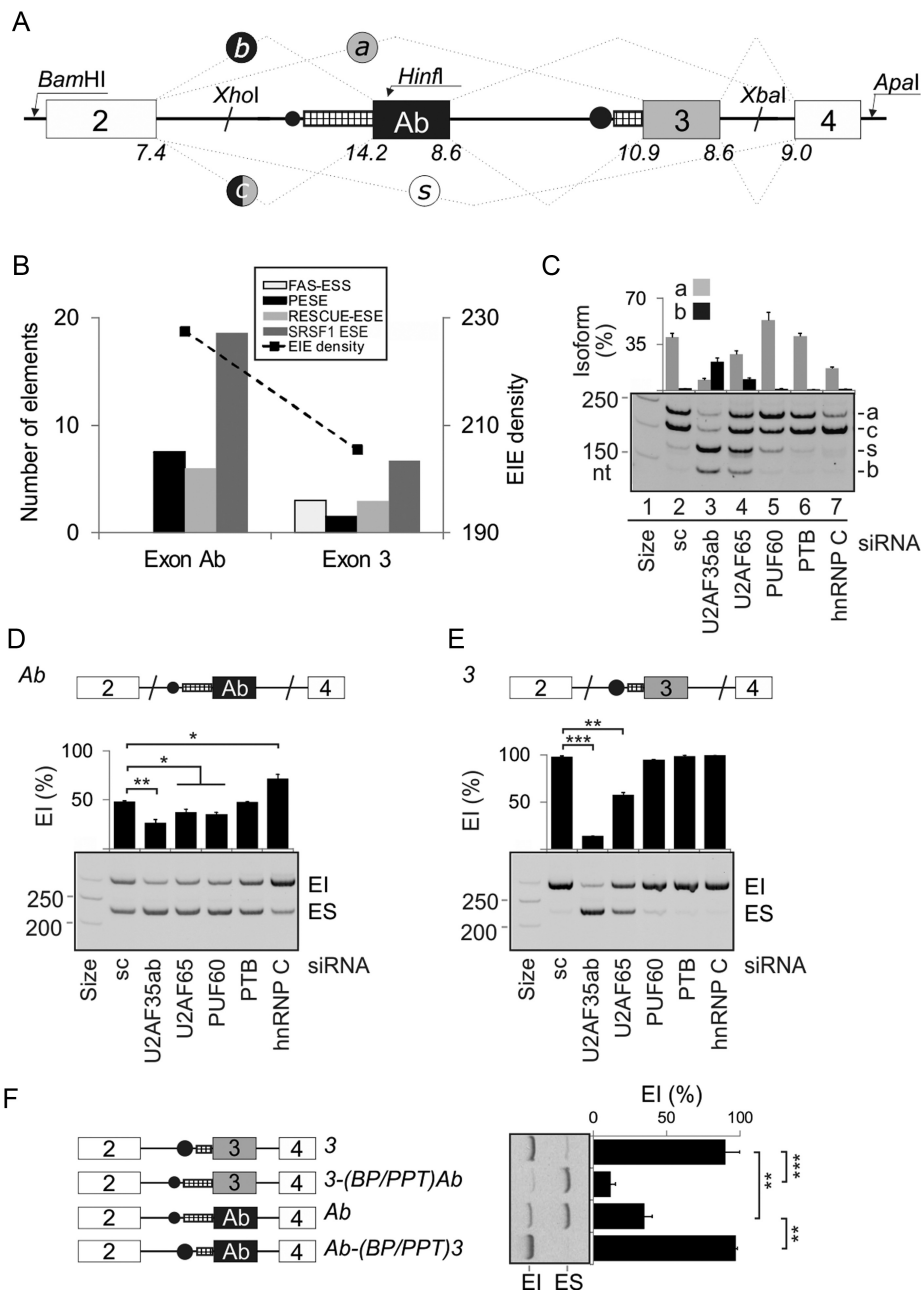
*U2AF1* exon Ab is repressed by the BP/PPT unit. (**A**) Alternative splicing of *U2AF1* and schematics of the 4-exon reporter. Exons are shown as boxes, introns as horizontal lines, spliced products (*a, b, c, s*) as dotted lines, exon Ab/3 BPs as black circles and their PPT as hatched rectangles. The size of black circles represents a predicted BP strength; the rectangle width denotes the length of PPT. Maximum entropy splice-site scores are below each exon-intron junction. (**B**) Density of predicted splicing enhancers in alternatively spliced *U2AF1* exons. For abbreviations, see Materials and Methods. (**C**) Regulation of alternative splicing of *U2AF1* by Y-binding proteins. Spliced products of the 4-exon reporter digested with *Hinf*I, which cuts intron 1–exon 2 junction and exon Ab but not exon 3 (20), are shown to the right. Depleted proteins are at the bottom, immunoblots are in Supplementary Figures S1 and S2. sc, a mixture of scrambled controls. HepG2 cells showed similar splicing patterns for each depletion. (**D** and **E**) Mean inclusion levels of alternatively spliced mid-exons in the mRNA. The 3-exon reporters are shown at the top, spliced products to the right and siRNAs at the bottom. EI, exon inclusion, ES, exon skipping. Error bars represent SDs of two transfections. Significant changes in panels D–F, 2H and Figure 3C are denoted by asterisks (*P< 0.05, **P< 0.01, ***P< 0.001). (**F**) Schematic representation of permuted constructs (*left panel*) and their exon inclusion levels in HEK293 cells (*right panel*). Their sequences are in Supplementary Table S2.

U2AF35 and U2AF65 are encoded by the *U2AF1* and *U2AF2* genes, respectively. Each gene is alternatively spliced, giving rise to highly similar protein isoforms (12,20). Alternative splicing of *U2AF1* generates two isoforms (U2AF35a and U2AF35b) encoded by tandem 67-nucleotide (nt) exons 3 and Ab (20) (Figure 1A). These exons arose by a duplication event that was followed by a relatively minor divergence maintained throughout vertebrate evolution (20). *U2AF1* transcripts that include or exclude both alternatively spliced exons contain stop codons and are downregulated by nonsense-mediated RNA decay (NMD) (12,20). Exons Ab and 3 encode a portion of the UHM (21), introducing just seven amino acid variants located in the RNP2 motif, a short disordered region containing phosphoserines, and an unusually long α-helix, also known as helix A or α2 (22,23). The UHM interacts with the UHM ligand motif (ULM) of U2AF65 (21–23) and provides a scaffold for two highly conserved C3H-type zinc finger domains (ZFs) that cooperatively bind RNA (23). The C-terminal serine/arginine-rich (RS) domain of U2AF35 is less conserved and is separated from ZF2 by a variable glycine linker (24,25). Both U2AF35a and U2AF35b can form heterodimers with U2AF65 that recognize highly overlapping sets of 3′ss, but selective knockdown of each isoform revealed transcripts and exons with isoform-specific responses, suggesting that their function in RNA processing is not equivalent (12,20,26). Moreover, although the abundance of endogenous U2AF35a was higher than U2AF35b in several tissues (20), exogenous expression of U2AF35a was lower than U2AF35b and endogenous U2AF35b levels were dramatically increased upon U2AF65 knockdown (12). Despite a growing evidence for a distinct function of U2AF35 proteins (12,20,26), molecular mechanisms leading to differential exon Ab/3 recognition have remained unknown. Although multiple contacts were identified between the UHM and ZFs in the yeast model (23), interactions between the dimorphic UHM in vertebrates and other U2AF35 domains are not fully understood.

Gene and exon duplications are principal events in evolution (27–29). If a duplicated exon is recognized by the spliceosome and introduces stop codons in the translational reading frame, mRNA surveillance such as NMD generally downregulates transcripts with both exons, including *U2AF1* (12,20). This will increase the relative abundance of transcripts containing either exon in the mRNA pool, forcing the cell to explore their function following mutation-driven diversification of duplicated regions. This process requires efficient repression or activation of duplicated exon copies, which can be achieved by steric interference, secondary structure, NMD or regulatory *trans*-acting factors (reviewed in (30)), but there is little understanding of molecular interactions between newly acquired mutations and spliceosome components that eventually lead to developmental stage-, environmental cue- or cell type-specific expression of new exons.

Here, we describe *cis-*acting elements and *trans-*acting factors regulating alternatively spliced *U2AF1* exons and identify similarities between the ‘mutually exclusive’ splicing of *U2AF1* and other U2AF-dependent tandem exon duplications. We also demonstrate that the differential expression of U2AF35a and U2AF35b requires interactions between U2AF65 and the α2/α6 helices of U2AF35.

## MATERIALS AND METHODS

### Cloning and mutagenesis

*U2AF1* reporter constructs containing exons 2, Ab, 3 and 4 were cloned into pCR3.1 by ligating PCR amplicons 1–3 (sized 655, 2075 and 274 nt) obtained with primers 1-F-BamHI and 1-R, 2-F-XhoI and 2-R-ApaI, and 3-F and 3-R-ApaI (Supplementary Table S1). Amplicons 1 and 3 contained internal XhoI and XbaI sites, respectively (Figure 1A). The 3-exon *U2AF1* minigenes (termed Ab and 3 according to their central exon) were cloned by ligating fragments amplified with primers PL4 (31) and 3Ea-XhoI or PL3 (31) and 3Eb-XbaI to XhoI/ApaI- or BamHI/XbaI-digested 4-exon constructs.

Wild-type U2AF35 and U2AF65 expression plasmids (pCI-neo, Promega) had an N-terminal Xpress tag (19). The U2AF35 construct was insensitive to the small interfering RNA (siRNA) U2AF35ab (19). Deletion constructs were created by overlap extension PCR using the same vector. A plasmid expressing FUBP1 (32) was a generous gift from Dr Sylvie Tuffery-Giraud, INSERM.

To create bicistronic constructs expressing various *U2AF1* segments, we prepared a green fluorescent protein (GFP)-expressing plasmid by inserting *GFP* into the BamHI/NotI site of pcDNA3.1His/Xpress (Invitrogen). The BglII/XbaI fragment of the plasmid DNA was inserted into the BglII/NheI site of pGL3-Luc (Promega). The luciferase (Luc) gene was removed together with the pGL3 SV40 promoter by a BglII/HpaI digest and replaced with tested CMV-U2AF35-SV40late poly(A) cassettes from the pCI-neo plasmids described above. For *in vitro* translation, we employed pcDNA3.1-U2AF35a and -U2AF35b plasmids as described (19). The pcDNA3.1His/Xpress-GFP was used to create hybrid ZF-GFP constructs for cotransfection studies with the plasmid expressing U2AF65. Each plasmid was propagated in *Escherichia coli DH5α*. Plasmid DNA was extracted with the GeneJET Plasmid Miniprep Kit (Thermo Scientific) and all plasmids were sequenced to confirm mutations and exclude undesired changes.

### Cell cultures, transfections and splicing assays

Human embryonic kidney (HEK) 293 cells were grown as described (33). Transient transfections with plasmids and siRNAs were performed with jetPRIME (Polyplus) according to manufacturer's recommendations. siRNAs are listed in Supplementary Table S1 or were described previously (19,33). Knockdown of heterogeneous nuclear ribonucleoprotein C (hnRNP C) was achieved by the HSS179304 siRNA (Invitrogen) (13). Cells were harvested 24–48 h after transfections with the indicated reporter constructs for RNA and western blot analyses. For RNA stability measurements, DRB (5,6-dichloro-1-β-d-ribofuranosylbenzimidazole; Sigma) and actinomycin D (Sigma) were added at a final concentration of 20 and 7.5 μg/ml, respectively, to replicates of HEK293 cell cultures for the indicated time points. Total RNA was extracted with TRI-reagent, treated with Turbo-DNase (Ambion) and reverse transcribed with the M-MLV reverse transcriptase (Promega) and the d(T)_20_VN primer. Exogenous transcripts were amplified for up to 28 cycles using primers PL3 and PL4. PCR products were separated using agarose or polyacrylamide gel electrophoresis and signal intensities of RNA products were measured as described (34).

### RNA pull-down assay

RNA pull-downs were carried out essentially as described (35). Briefly, 500 pmols of synthetic 25-mers corresponding to the wild-type ESL and its mutated version (Supplementary Table S1) were treated with 5 mM sodium *m*-periodate and bound to adipic acid dihydrazide agarose beads (Sigma). Beads with bound RNAs were washed three times in 2 ml of 2 M NaCl and three times in buffer D (20 mM HEPES–KOH, pH 7, 6.5% v/v glycerol, 100 mM KCl, 0.2 mM EDTA, 0.5 mM dithiothreitol), incubated with HeLa nuclear extracts and buffer D with heparin at a final concentration of 0.5 mg/ml. Unbound proteins were washed five times with buffer D. Bound proteins were separated on 10% sodium dodecyl sulphate polyacrylamide gel electrophoresis (SDS-PAGE) or gradient NuPAGE 4–12% gels, stained with the Coomassie blue and/or blotted on to nitrocellulose membranes. Gel fragments specific for tested RNAs were digested with trypsin and subjected to tandem MS using a Bruker ultraflex III MALDI-TOF/TOF at the Proteomic Technology Facility of the University of York.

### Immunoblotting

Western blot analysis was carried out as described (19) using antibodies against FUBP1 (GeneTex, GTX104579), FUBP2 (also known as KHSRP or KSRP; Bethyl Laboratories Inc., A302-021A-T), His-tag (Qiagen, 34660), PTBP1 (36), SRSF3 (Sigma, WH0006428M8), TIA1 (Proteintech, 12133-2-AP), TIAR (Cell Signaling Technology Inc. D32D3), hnRNP E1/E2 (Sigma,R4155), MBNL1 (Sigma, M3320), DHX36 (ABcam, ab70269), CHEK2 (Cell Signaling Technology Inc., D9C6), PUF60 and hnRNP C (generous gifts from Professor Adrian Krainer, CSHL, and Professor Gideon Dreyfuss, University of Pennsylvania, respectively). Antibodies against U2AF35, U2AF65, RBM39, Xpress and tubulin were described previously (12).

### U2AF35 degradation pathways

Plasmids expressing U2AF35 isoforms (120 ng/ml) were individually cotransfected with pGFP (50 ng/ml) into HEK293 cells. The proteasome inhibitor MG132 (Sigma) was added 36 hrs after plasmid transfections at a final concentration of 10 μM. Cell lysates were separated by SDS-PAGE and immunoblots were successively incubated with antibodies against the Xpress tag, U2AF35, GFP and U2AF65. The lysosomal inhibitor NH_4_Cl was added to a final concentration of 30 mM to HEK293 cells cotransfected with U2AF35a or U2AF35b and U2AF65. Blots were incubated with the Xpress antibody.

### Cell-free U2AF35 synthesis

*In vitro* translation reactions were carried out using the TNT^®^ Quick Coupled Transcription/Translation System (Promega) according to the manufacturer's recommendations. Twenty five microliter-reactions contained 20 μl of TNT Master Mix, 1 μl of [^35^S]-methionine (1000 Ci/mmol at 10 mCi/ml), 50 ng of a control luciferase plasmid and 600 ng of plasmids expressing *U2AF1* isoforms. Reactions were incubated for 90 min at 30°C and their aliquots were loaded on to NuPAGE 4–12% Bis–Tris gels (Invitrogen). Gels were dried and exposed to phosphorimager screens. Signal intensity was measured with ImageQuant TL.

### Branch point mapping

HEK293 cells were grown in DMEM with or without *DBR1* siRNAs (Supplementary Table S1) and harvested 72 h after (mock) transfection for RNA extraction. The final concentration of each duplex was 40 nM. *DBR1* encodes a debranching enzyme that hydrolyzes 2′-5′ branched phosphodiester bonds, converting lariats into linear molecules for degradation (37). A lack of debranching activity *in vivo* leads to accumulation of excised lariat introns. Total RNA was extracted using TRI-reagent and treated with DNase. One microgram of purified RNA was reverse transcribed with the SuperScript™ III cDNA synthesis kit (LifeTechnologies) and primer R1 (Supplementary Table S1). For exon Ab, the first-strand cDNA was amplified with outer primers F1 and R1 in the first round of PCR, which was divided into multiple second rounds of PCR with inner primers F2 and R2 (Supplementary Table S1). For exon 3, we employed primers R1/F1-3 and R2/F2-3. Each step was carried out at several annealing temperatures. Amplicons were gel-purified, ligated into pGEM-T Easy (Promega) and sequenced.

RNA-Seq data generated from cultures treated with or without siRNAs targeting *U2AF1* isoforms (ArrayExpress accession number E-MTAB-2682) were searched for 15- and 20-nt sequence strings at the 5′ss of *U2AF1* intron 2 (three mismatches allowed). In addition, we analysed ENCODE RNA-Seq data from 14 cell lines sequenced using Illumina GA and GAII (38) and Illumina Body Map data of 16 different human tissues sequenced using Illumina HiSeq 2000.

### Relative abundance of *U2AF1* isoforms

We employed the FirstChoice human total RNA survey panel with 20 different tissues, each containing a pool of RNAs from different donors (LifeTechnologies). In addition, we used total RNA extracted from the indicated cell lines. Rodent tissue samples were removed from identical organ locations (*n* = 7) of C57BL/6 mice and Wistar, SHR24 and Sprague-Dawley rats. All animals were females aged 4 weeks at sampling. Animals were sacrificed by cervical dislocation, conforming to regulations of a local ethics committee. Organs were frozen immediately upon collection in liquid nitrogen and stored at −80°C for subsequent total RNA extraction. All RNA samples were reverse transcribed using oligo-d(T) primers and complementary DNAs were amplified with PCR primers described previously (20) for 26 cycles. Rat samples were amplified with a reverse primer E6R (Supplementary Table S1).

### Bioinformatic and statistical analyses

Densities of auxiliary splicing motifs previously defined as FAS-ESSs (39), PESS/PESEs (40), RESCUE-ESEs (41), putative SR proteins ESEs (42) and EIEs (43) were calculated as described (44). BPs were predicted using a support vector machine (SVM) (45) or human-mouse (HM) comparison (46) algorithms. The PPT length was determined by the SVM-BP tool (45). Maximum entropy splice-site scores were calculated as described (47). RNA secondary structures were computed using phylogenetic stochastic context-free grammar (48) and also mfold (49) and RNAbows (50) to identify stable structures across conserved *U2AF1* regions. The PU (probability of unpaired) values were computed as described (51) using human reference sequences of the U2AF-regulated homologous exons (Table 1), their flanking introns and additional 30 nt in each direction. Descriptive statistics and correlation coefficients were computed using Stat200 (Biosoft, UK).

**Table 1.** 3′ splice site organization of U2AF-regulated tandem homologous exon

Gene	Exon (isoform)	U2AF-mediated	Reference	BP SVM score	PPT length^a^	AGEZ
*FYN*^b,e^	7a (FYNB)	Repression	Figure S20A,C	0.66	43	61
	7b (FYNT)	Activation	Figure S20A,C	1.01	11	35
*TPM1*^e^	6a	Activation	(12)	1.21	6	104
	6b	Repression	(12)	1.21	67	88
*TPM2*^e^	6a	Activation	(12)	2.34	16	34
	6b	Repression	(12)	1.75	106	131
*U2AF1*^e^	Ab (U2AF35b)	Repression (or less efficient activation)	Figure 1C–E	Not predicted	25	31
	3 (U2AF35a)	Activation	Figure 1C–E	Not predicted	9	21
*CALU*^c,e^	3a	Repression	E-MTAB-2682^d^	1.39	27	48
	3b	Activation	E-MTAB-2682	1.29	12	35
*ACOX1*	3a	Activation	E-MTAB-2682	0.98	19	24
	3b	Repression	E-MTAB-2682	1.31	10	59
*MAPK14*^e^	9a	Repression	E-MTAB-2682	0.16	32	61
	9b	Activation	E-MTAB-2682	−0.47	8	56
*P4HA1*^e^	10a	Activation	E-MTAB-2682	0.96	7	44
	10b	Repression	E-MTAB-2682	1.03	22	26

^a^PPT length (nt) is for predicted BPs unless determined experimentally, as shown in Supplementary Figures S8 and S12.

^c^*FYN* exon 7a is a younger copy of exon 7b (118). These exons encode functionally distinct FYNT and FYNB proteins regulated by U2AF (summarized in Supplementary Figure S20B).

^c^Activation of alternatively spliced *CALU* exon 3b (also known as exon 4) was associated with a promotion of distal transcription initiation site in HEK293 cells depleted of U2AF35 (Supplementary Figure S21).

^d^Accession number of RNA-Seq data for U2AF35 knockdowns.

^e^Transcripts containing Evofold-detected hairpins.

## RESULTS

### Positive and negative regulation of alternatively spliced *U2AF1* exons Ab and 3

*U2AF1* exon Ab has a longer PPT than exon 3 (Figure 1A), which has been associated with better recognition of vertebrate exons (52,53), lacks splicing silencers and has a higher density of splicing enhancers, including an excess of predicted binding sites for SR proteins such as SRSF1 (Figure 1B). The weaker 3′ss of exon 3 is not compensated by a stronger 5′ss, yet exon Ab is included in the *U2AF1* mRNA less efficiently than exon 3 (20). Why is the more optimal exon Ab repressed *in vivo*?

To begin to answer this question, we first prepared a 4-exon splicing reporter with exons Ab and 3 in the middle (Figure 1A). Transfection of the wild-type construct into HEK293 cells and visualization of exon Ab inclusion using HinfI digests of spliced products confirmed the lower abundance of *U2AF1b* than *U2AF1a* (Figure 1C), thus recapitulating exon inclusion levels observed *in vivo*. As expected for exogenous, ‘NMD-immune’ RNAs, minigene products containing (*U2AF1c*) or lacking (*U2AF1s*) both exons were more abundant than in endogenous transcripts (Figure 1C).

The extended PPT of exon Ab may bind other PPT-binding proteins that compete with U2AF65 (13,54–56). Transfection of this construct into cells individually depleted of U2AF35, U2AF65 and a subset of Y-binding proteins (Supplementary Figure S1) showed an increased relative abundance of *U2AF1b* in cells depleted of U2AF35 or U2AF65 (Figure 1C, lanes 2–4). In contrast, knockdown of a U2AF65-related protein PUF60 activated *U2AF1a* (lane 5) while hnRNP C depletion stimulated inclusion of both exons (lane 7). Transfection of 3-exon minigenes with exons Ab or 3 in the middle confirmed that exon Ab was less dependent on each U2AF subunit than exon 3 and was promoted by PUF60 (Figure 1D and E). This exon was repressed by hnRNP C, consistent with direct competition between U2AF65 and hnRNP C (13), although exon 3 was activated at higher siRNA concentrations (Supplementary Figure S2).

To evaluate the extent to which PPTs and predicted BPs contribute to inclusion levels of exon Ab and 3, we exchanged 47-nt segments (position −4 to −50) upstream of their 3′ss in 3-exon minigenes and examined spliced products of the resulting hybrids (Figure 1F, Supplementary Table S2). The BP/PPT of exon Ab placed upstream of exon 3 conferred exon skipping whereas the BP/PPT of exon 3 increased exon Ab inclusion.

We conclude that (i) the information required for exon Ab repression is encoded by exon Ab and/or flanking introns (Figure 1C, lane 2), (ii) the BP/PPT unit of exon 3 is a more efficient exon activator than that of exon Ab, despite a longer PPT of the latter (Figure 1A, F), (iii) the BP/PPT of exon Ab is sufficient to inhibit its inclusion in the mRNA (Figure 1F) and (iv) alternative splicing of *U2AF1* is regulated by its own product and other Y-binding proteins (Figure 1C–E).

### Identification of branch sites of alternative *U2AF1* exons

**Figure 2.**
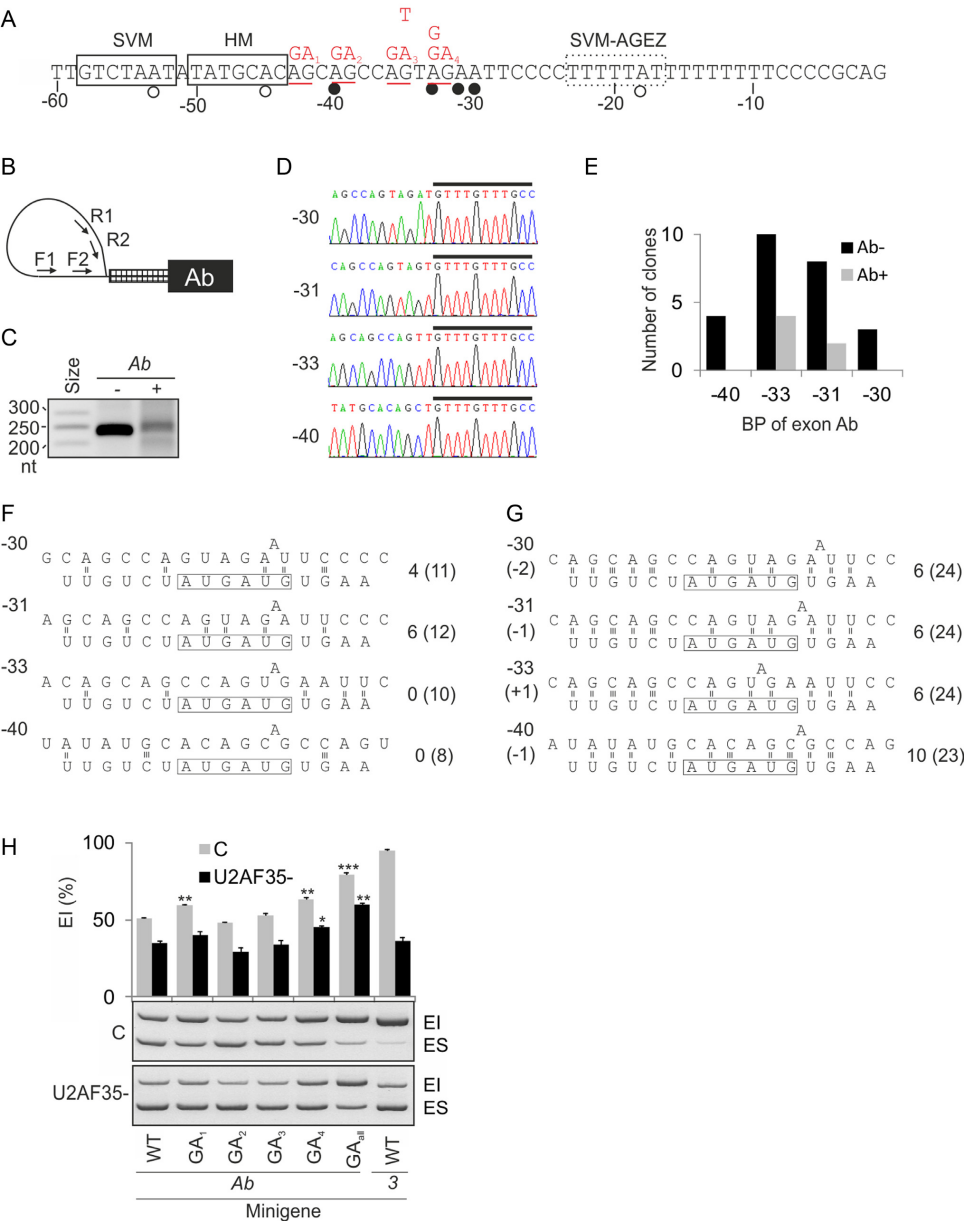
*U2AF1* exon Ab employs multiple noncanonical branch points. (**A**) Nucleotide sequence upstream of exon Ab. Predicted and experimentally determined BPs are shown by open and closed circles, respectively. Boxes denote BP heptamers predicted using the support vector machine (SVM) (45) or the comparative human-mouse (HM) (46) algorithms; a dotted box denotes a weak SVM BP heptamer (SVM score of −0.71) predicted with the AGEZ filter. The distance relative to the 3′ss of exon Ab is shown at the bottom. Mutations introduced in the Ab reporter construct (Figure 1D) are in red at the top. (**B**) Primers for BP mapping (Supplementary Table S1). (**C**) Lariat introns amplified from DBR1-depleted HEK293 cells mock-transfected (−) or transfected (+) with the Ab reporter. (**D**) Sequence chromatograms of exon Ab lariats. Distances of BP adenines relative to the 3′ss are to the left. A black rectangle denotes the 5′ end of *U2AF1* intron 2. (**E**) Exon Ab BP usage in cells (mock)-transfected with the Ab reporter. (**F**) Predicted canonical base-pairing interactions of each BP sequence with the U2 snRNA. The BP-interacting motif of U2 snRNA is boxed. The number of predicted hydrogen bonds between the AUGAUG box of U2 and the pre-mRNA is to the right. The number of predicted hydrogen bonds between the pre-mRNA and extended single-stranded region of U2 snRNA is in parentheses. (**G**) Predicted alternative base-pairing for each BP. Shifts of the U2 snRNA sequence towards the 5′ (−) or 3′ (+) intron ends are shown in parentheses to the left. Hydrogen bonds are numbered to the right as in panel F. (**H**) Exon inclusion levels of AG mutants in U2AF35ab siRNA-treated (U2AF35-) and mock-treated (C) cells. Error bars denote SDs of duplicate transfections. Mutations are shown in panel A.

To determine if the BP strength contributes to inclusion levels of exon Ab and 3, we first examined data from large-scale BP mapping studies (57–59). They reported BPs for ∼20% of human exons, but did not identify any BP of exon Ab. We next searched our own RNA-Seq data for samples depleted of *U2AF1a* (12) for reads containing the 5′ end of intron 2 and lacking exon 2 ends, however, samples enriched for *U2AF1b* were not informative either. Prediction of exon Ab BPs using HM (46) and SVM (45) algorithms produced distinct BP locations, each with at least one AG dinucleotide in the AG exclusion zone (AGEZ) between the predicted BP and 3′ss (Figure 2A). AGEZs contain the majority of BPs (45,59), but AGs in AGEZs are selected by the splicing machinery as 3′ss only if located >8–12 nt downstream of genuine BPs (45,60,61), suggesting that the BP predictions were incorrect. The AGEZ-filtered SVM prediction produced only a low-confidence BP with a negative SVM score (Figure 2A).

BP mapping in DBR1-depleted HEK293 cells (mock)-transfected with the Ab minigene (Figure 2B) showed ∼250 nt fragments in each culture (Figure 2C). Sequencing of 31 subclones revealed a cluster of four BP adenines close to each other between position −40 and −30 relative to exon Ab 3′ss, just upstream of the long PPT (Figure 2D and E). No BPs had a canonical uridine at position −2 relative to BP adenine (Figure 2A, D and E). BP at position −31 (BP-31) had the highest number of predicted hydrogen bonds with the BP-interacting region of U2 snRNA (Figure 2F) although it was not used most frequently. Interestingly, predicted base-pairing interactions were much stronger when the BP and surrounding sequences were shifted by 1 or 2 nucleotides (Figure 2G).

The weak BP cluster of exon Ab accommodates an unusual set of four AGs (underlined in Figure 2A). To test their importance for exon Ab inclusion, we mutated each AG in the Ab minigene. Mutations of the first and fourth AG increased exon Ab inclusion, with an additive effect for their combination, which was mirrored in cells lacking U2AF35 (Figure 2H). The highest inclusion was found for mutation of the AG most proximal to the 3′ss, which created an optimal BP consensus UNA_-32_ (62) next to BP-33. Elimination of the strongest BP-33 by the A>G mutation and the *in situ* improvement of the BP sequence consensus (mutation −35G>T) had virtually no effect (Figure 1A and Supplementary Figure S3), consistent with simultaneous recognition of weak competing BPs that compensate each other.

To map BPs of exon 3, we employed primers for both intron 2 and intron Ab since exon Ab is not a dominant exon. Sequencing of a single product implicated adenines −25 and −27, just downstream of a predicted BP (Supplementary Figure S4), confirming the latter BP identified by RNA-Seq (59).

Taken together, exon Ab has an atypical constellation of weak, closely spaced BPs located immediately upstream of its long PPT. None of these BPs were predicted computationally and they all lacked the human UNA consensus, suggesting that they require compensatory *cis*-elements and/or *trans*-acting factors.

### A conserved, FUBP1/2-bound motif upstream of the BP cluster regulates exon Ab

**Figure 3.**
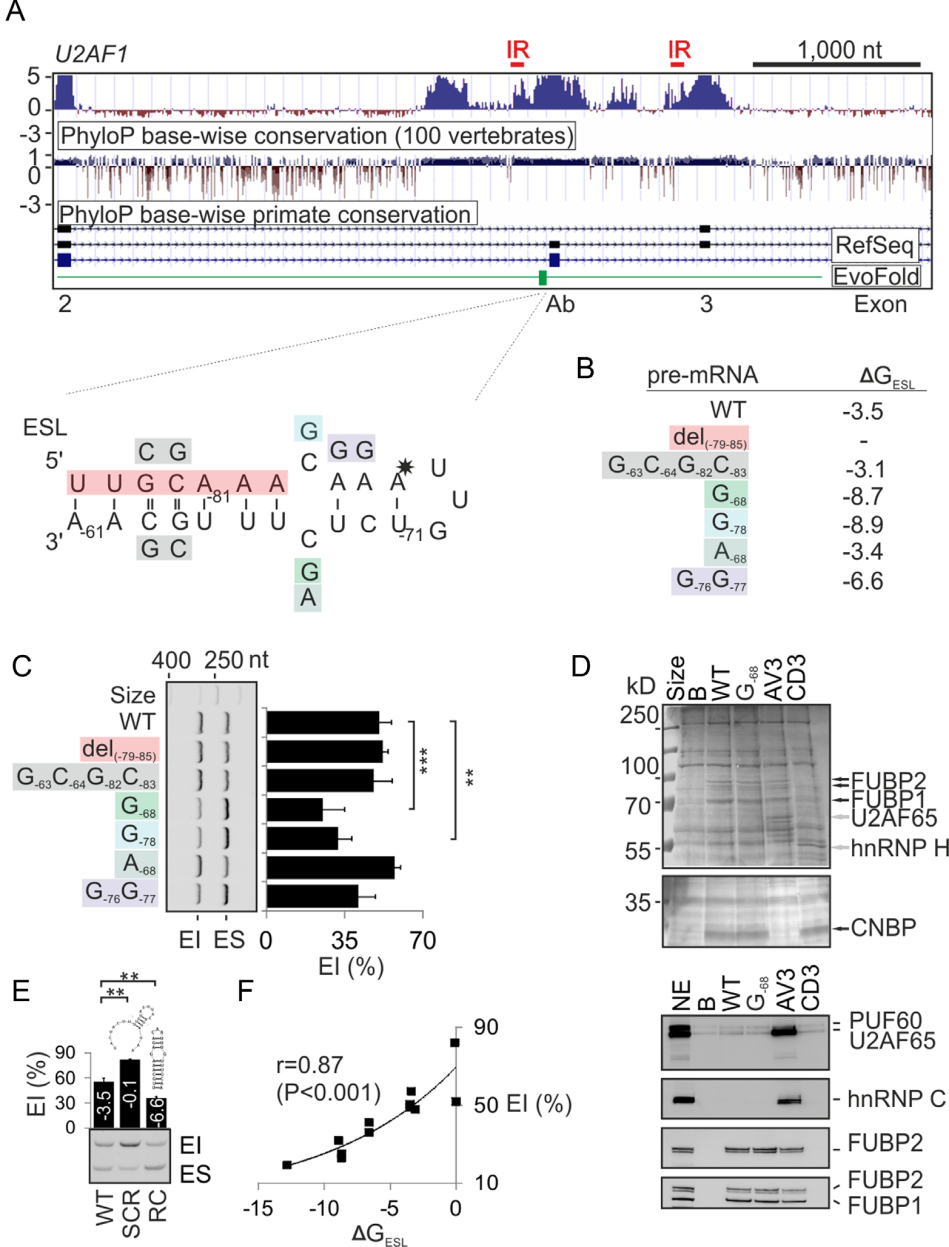
Internal loop of the predicted ultraconserved hairpin upstream of the BP cluster controls exon Ab inclusion. (**A**) Sequence conservation across alternatively spliced *U2AF1* exons Ab and 3. PhyloP conservation UCSC tracts are followed by the RefSeq annotation of *U2AF1* exons (numbered at the bottom). ESL (in green) is expanded in the lower panel; mutations are colored. ESL nucleotide positions are numbered according to their distance from the 3′ss of exon Ab. Asterisk indicates an A>G substitution conserved in many tetrapods (Supplementary Figure S5) and predicted to stabilize the hairpin. Red horizontal bars at the top denote location of the longest inverted repeats (IR) in the conserved regions (Supplementary Figure S9). (**B**) Predicted free energies (ΔG_ESL_) of the wild-type (WT) and mutant RNAs (kcal/mol). (**C**) Exon inclusion levels of ESL-mutated pre-mRNAs. Columns represent means and error bars SDs of two transfections of the Ab reporter (Figure 1D). (**D**) RNA pull-down assay (*upper panel*) and immunoblots with the indicated antibodies (*lower panel*). B, beads only, G_-68_, mutated synthetic RNA (Supplementary Table S1). AV3 and CD3 are control RNAs described previously (19,35). NE, nuclear extract. CNBP (also known as ZNF9) is a CCHC-type zinc finger nucleic acid binding protein identified by mass spectrometry. (**E**) Exon inclusion (%) in the mRNA from WT and mutated Ab constructs. Scrambled (SCR) and reverse complement (RC) sequences were gactacttttctacttacaggataa and ttgcaaagagacaatttgtttgcaa, respectively. Their predicted energies (kcal/mol) and schematic secondary structures are as indicated. (**F**) Positive correlation between predicted ΔG_ESL_ and exon Ab inclusion levels of 11 different ESL reporters.

Both alternatively spliced *U2AF1* exons are preceded by two regions highly conserved in vertebrates, with a maximum conservation at ∼100 nt and ∼500 nt upstream of their 3′ss (Figure 3A). The 3′ region upstream of exon Ab accommodates an EvoFold-detected (48) stem loop (termed ESL), occupying positions −61 to −85 relative to the 3′ss (Figure 3A and Supplementary Figure S5). EvoFold employs a stochastic context free grammar algorithm involving covariation to identify functional RNA structures (48). To test the importance of ESL for exon Ab recognition, we introduced a series of mutations in the Ab reporter predicted to destabilize (7-nt deletion of the 5′ stem) or stabilize (C_-68_>G and C_-78_>G substitutions) the hairpin or maintain self-complementarity of the stem (a G_−63_C_−64_ swap, Figure 3B and C). Transfections of mutated constructs into HEK293 cells revealed that the ESL stabilization diminished exon inclusion, indicating that exon Ab is promoted by interactions involving unpaired cytosines in the predicted internal loop.

RBPmap (63) predictions for ESL and flanking sequences suggested that the upper part of ESL may contain binding sites for PTBP1, TRA2B and SRSF3 (Supplementary Figure S6), but tested mutations in putative PTBP1 (A_−68_) and TRA2B (G_−76_G_−77_) binding motifs had little effect on exon Ab splicing (Figure 3C). To identify proteins that bind ESL, we carried out RNA pull-down assays with a synthetic wild-type RNA and its mutated version (C_−68_>G) that reduced exon Ab inclusion (Figure 3C and D). Comparison of their binding patterns with control RNAs, followed by mass spectrometry and immunoblotting, identified a specific interaction with the far-upstream element-binding proteins 1 and 2 (FUBP1 and FUBP2). Overexpression and depletion of FUBP1 slightly reduced and increased exon Ab inclusion of the ESL G_-68_ mutants, respectively, but a loop mutation changing one of the predicted binding sites for FUBP1 (UU_-73_GU>UAGU) (64) had no effect (Supplementary Figure S7).

To further test the role of ESL in exon Ab recognition, we replaced the complete hairpin with its scrambled and reverse complement versions in Ab minigenes (Figure 3E). The scrambled form maintained the same base composition but introduced an unstable structure, thus addressing a possibility that the deletion of lower ESL stem (del_-79–85_ in Figure 3A–C) could still maintain intramolecular base-pairing that fully support ligand interactions of the upper part. By contrast, the reverse complement version was predicted to maintain the overall structure while replacing the identity of all unpaired residues. The former mutation significantly increased exon Ab inclusion whereas the latter mutation reduced inclusion levels (Figure 3E), while further ESL hyperstabilization by mutation G_−76_G_−68_ yielded a similar effect (Supplementary Figure S7). Importantly, exon Ab inclusion levels and predicted free energies of our wild-type and mutated constructs showed significant correlation (Figure 3F), arguing for a major role of ESL stabilities in regulating exon Ab levels *in vivo*. The ESL importance was further supported by a pair-wise alignment of exons Ab and 3 together with their upstream conserved regions, showing a lack of both ESL and exon Ab BP sequences upstream of exon 3 (Supplementary Figure S8), suggesting that these motifs have not evolved independently.

In insects, mutually exclusive exon splicing has been linked to conserved base-pairing interactions between docking and selector sites adjacent to constitutive and variant exons (65,66). Interestingly, RNA secondary structure predictions with conserved sequences upstream of exons Ab and 3 revealed that formation of the most stable structures consistently involved the longest inverted repeats in this region (Figure 3A and Supplementary Figure S9). These inverted repeats are of similar length and location to those implicated in mutually exclusive splicing in insects (65,66), tentatively looping out exon Ab and contributing to its repression. They are devoid of any natural DNA variants, which are also absent in the ESL and the BP sequences of exon Ab (Ensembl ENSG00000160201). Surprisingly, neither deletions of their stem nor Ab/3 exon swaps of their central, most stable portion in inverted (mutation 1 and 2) or direct (mutation 3 and 4) orientations revealed any alterations of exon inclusion levels in our reporters (Supplementary Figure S9).

We conclude that exon Ab usage is tightly controlled by the ESL stability and that sufficient U2AF35b expression requires interactions between unpaired ESL positions and their ligands. The ESL is bound by FUBP1/2 that may potentially help to enforce correct ESL folding through their helicase activities.

### Identification of exon *cis*-elements and SR proteins that control alternative splicing of *U2AF1*

**Figure 4.**
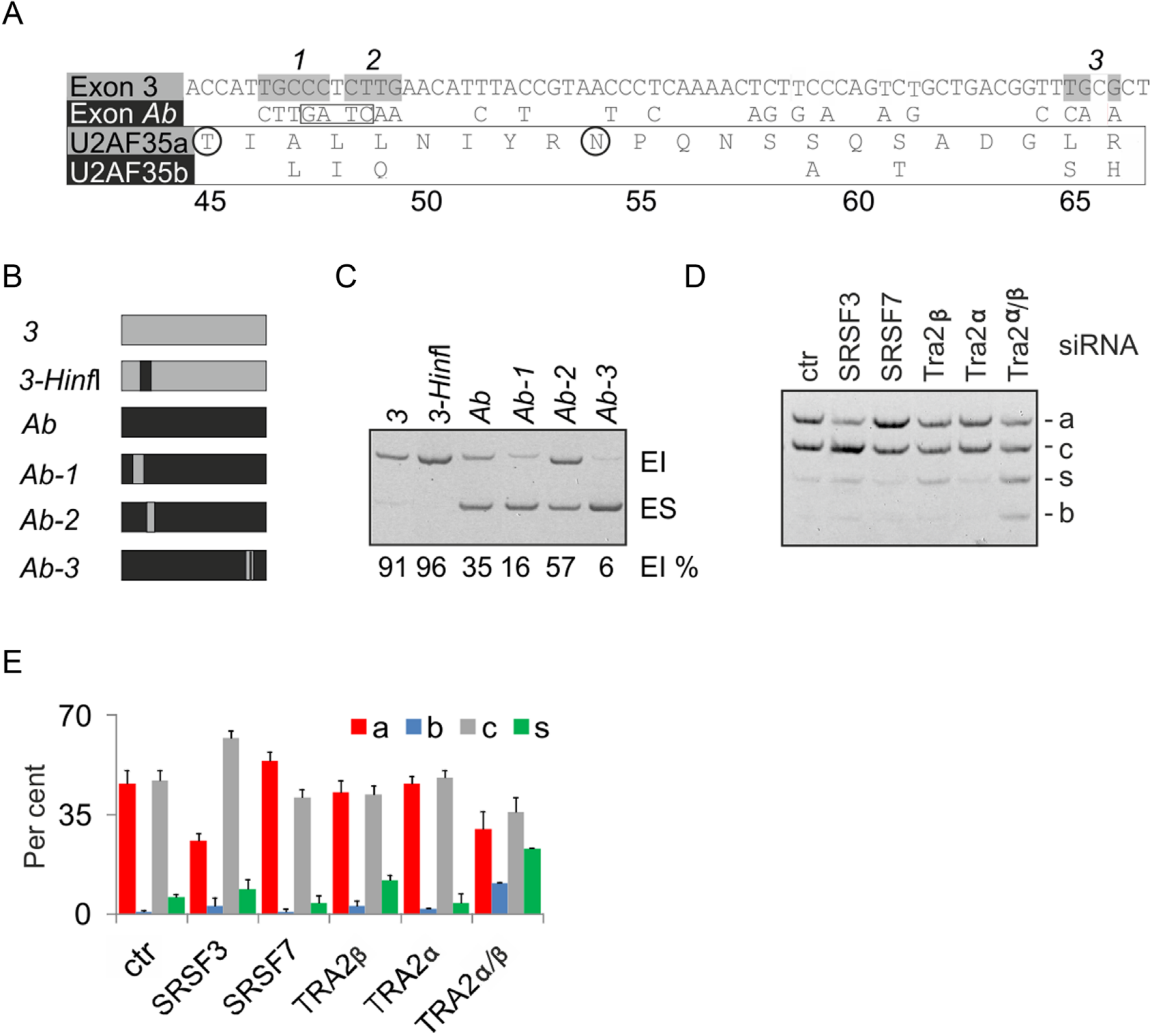
Identification of exonic variants that influence inclusion of alternative *U2AF1* exons. (**A**) Sequence differences between alternatively spliced *U2AF1* exons and U2AF35 isoforms. Amino acids are numbered at the bottom. Exon 3 segments introduced in exon Ab (numbered at the top) are highlighted in gray; the HinfI site is boxed. Homologous residues that interact with S43 of the U2AF35 ZF1 and K169 of the U2AF35 ZF2 in the yeast model (23) are circled. (**B**) Schematics of permuted middle exons in minigene Ab (black) and 3 (gray). Their full sequences are in Supplementary Table S3. (**C**) Exon inclusion levels of reporters shown in panel (B). (**D**) Splicing pattern of exogenous *U2AF1* transcripts in cells lacking the indicated SR(-like) proteins. Concentrations of siRNAs were as described (19). Ctr, control siRNA. Designation of *U2AF1* mRNA isoforms is as in Figure 1. (**E**) Isoform quantification for panel D. Error bars are SDs of two transfections.

To test if exonic variants contribute to exon Ab repression, we examined splicing of exon Ab/3 hybrid reporters (Figure 4A and B; Supplementary Table S3). Exon Ab was most promoted by exon 3 sequences that encode the U2AF35a RNP2 motif, as illustrated by mutation Ab-2 (Figure 4B, C). This Ab-to-3 swap changes glutamine 49 to leucine and creates a GAA trinucleotide, one of the most potent exonic splicing enhancer (33,67,68). The remaining insertions of exon 3 segments to exon Ab were closer to splice sites and promoted exon skipping. Exon inclusion was also slightly improved by introducing the exon Ab-specific HinfI site in the equivalent position of exon 3.

To identify additional *trans-*acting factors that regulate alternatively spliced exons Ab and 3, we individually depleted HEK293 cells of a subset of SR proteins, well-known splicing regulators (69), and examined the splicing pattern of 4-exon minigenes. Depletion of SRSF3 and co-depletion of Tra2α and Tra2β promoted *U2AF1b*, with SRSF3 knockdown dramatically stimulating U2AF35*c* (Figure 4D and E). In contrast, a lack of SRSF7 increased *U2AF1a*. SRSF1 knockdown did not significantly alter the *U2AF1a/b* mRNA ratio (data not shown). In cells depleted of Tra2 proteins, exon 3 was preferentially skipped. The same direction of exon Ab/3 usage was observed for endogenous *U2AF1* (except for isoforms targeted by NMD), with a significant correlation of *U2AF1b/U2AF1a* ratios between endogenous and exogenous transcripts (*r* = 0.66; *P*< 0.01).

### Identification of U2AF-regulated tandem exons controlled by SRSF3

Examination of our RNA-Seq data (12) for altered usage of mutually exclusive tandem homologous exons (listed in (70)) revealed a set of 8 exon pairs responsive to U2AF35 knockdown, in which one homolog was activated and the other was repressed (Table 1). To test their functional and structural similarities to exon Ab/3, we first examined their usage in independent depletions of each U2AF subunit, U2AF-related paralogs and other Y-binding proteins, including SRSF3. This analysis confirmed the antagonism of U2AF and PUF60 and the synergism between U2AF and RBM39 (Supplementary Figure S10A-D) (12), both proteins structurally related to U2AF65 (21). Interestingly, it also revealed that in most cases, the SRSF3 knockdown increased the relative abundance of transcripts that contained both homologous exons (Supplementary Figure S10B), indicating that in most pairs, SRSF3 is required for repression of a single homolog. Unlike other SR proteins, SRSF3 binding sites are Y-rich, with a core CNUC motif (71,72), suggesting that the functional affinity of U2AF-dependent exon homologs for SRSF3 could be explained by RNA binding. Examination of published ultraviolet crosslinking and immunoprecipitation (CLIP) data for Srsf3 and other SR proteins (71,72) revealed significant binding to *U2af1* exons Ab/3 as well as other homologous exon pairs. However, the Srsf3 CLIP tags did not extend into exon Ab BPs or ESL, although they were mapped to the BP/PPT of the U2AF-activated exon 3 (Supplementary Figure S10E). Nevertheless, the Srsf3 crosslinking events were present also near BPs of a U2AF-repressed exon in *Tpm1*. Taken together, U2AF-regulated duplicated exons showed preferential responses to Y-binding proteins SRSF3, PUF60 and RBM39.

### Organization of U2AF-regulated 3′ splice sites of duplicated exons

**Figure 5.**
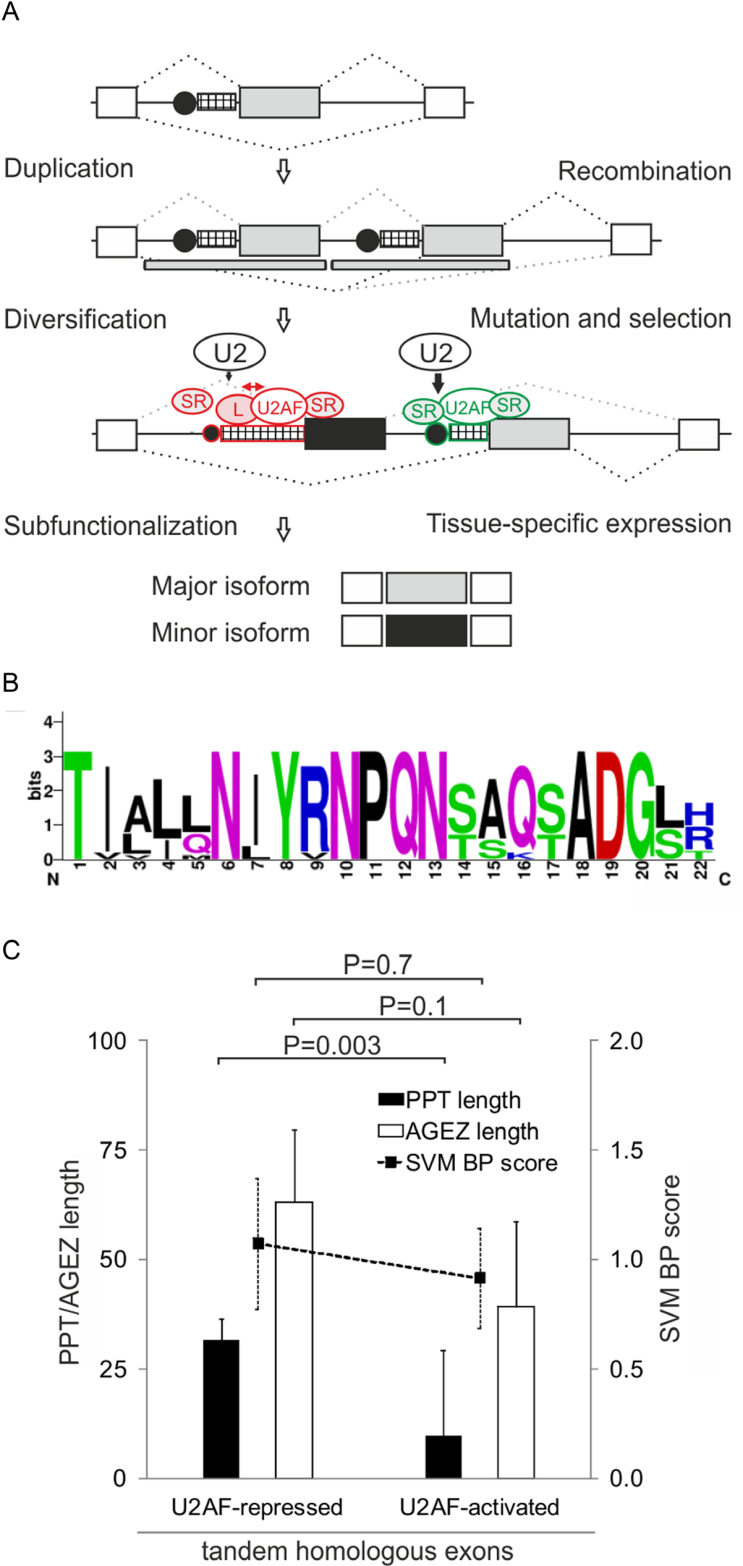
A role for BP/PPT interactions in subfunctionalization of tandem homologous exons. (**A**) BP/PPT ligands as repressors of duplicated exons. For BP/PPT symbols, see legend to Figure 1A. Ancestral and replica exons are denoted by gray boxes; flanking exons by white boxes; duplicated DNA segments by gray rectangles below; a repressed exon homolog by a black box; splicing by dotted lines. Exon-inhibiting and -stimulating motifs or splicing factors are shown in red and green, respectively. Red arrow signifies competition between U2AF and other PPT ligands (L), which may also bind U2 snRNP components, thus inhibiting U2 binding to the BP (black vertical arrows). (**B**) Pictogram representation of protein sequences encoded by exons Ab and 3 from 12 species, ranging from *D. melanogaster* to *H. sapiens*. Exon positions are numbered at the bottom. (**C**) Average PPT/AGEZ length and SVM BP scores of U2AF-responsive tandem exon homologs. Two-tailed *P*-values (t-test) are shown at the top.

The majority of invertebrate tandem exon duplications associated with mutually exclusive splicing resulted from homologous recombination (HR) events that engaged the upstream intron in each case (73). A HR-mediated duplication of the *U2AF1* intron 2–exon 3 segment (Figure 5A) would also explain the mutually exclusive splicing of exons Ab and 3 as well as the existence of the two regions of vertebrate conservation, which are located at a similar distance from their 3′ss (Figure 3A) and share significant sequence identity (Supplementary Figure S8). Although the least diverged exonic sequence encoding the almost invariant YRNPQN motif of the UHM (Figure 5B) may also constitute a favorable HR crossover region, the two exons share the 5′ss consensus (Supplementary Figure S8), arguing for a HR breakpoint further downstream. Importantly, comparison of duplicated exon pairs (Table 1) showed that exons activated by U2AF had invariably shorter PPTs than U2AF-repressed exons (or less efficiently activated, as in *U2AF1;* Figure 5C). To test if the differential PPT length alters their overall capacity for ligand interactions, we examined their base-pairing potential by computing PU (probability of unpaired) values, which estimate RNA singlestrandedness using the equilibrium partition function (51). Most intronic positions upstream of 3′ss of U2AF-repressed homologous exons exhibited significantly higher PU values than those upstream of U2AF-activated counterparts (Supplementary Figure S11A). Their mean was even higher than that reported for experimentally determined intronic splicing regulatory motifs (Table 2; 0.387 versus 0.351 in (51)) or for all U2AF-repressed exons identified globally (12). The higher probability of unpaired interactions was associated with an excess of pyrimidines and depletion of purines, particularly cytosine and guanine, respectively (Supplementary Figure S11B). In contrast, the first ∼10 positions of the exon tended to be more single-stranded for U2AF-activated exons (Supplementary Figure S11C) while the PU values downstream were similar (Table 2, Supplementary Figure S11D).

**Table 2.** Mean PU values for U2AF-repressed and -activated duplicated homologous exons

	Upstream of 3′ss^a^	Downstream of 5′ss^a^
U2AF-repressed exons	0.387	0.177
U2AF-activated exons	0.146	0.194
*P*-value	*P*<10^−16^	*P*> 0.1

^a^Mean PU values were calculated by averaging position-specific values computed for human genomic reference sequences (hg19) in segments shown in Supplementary Figure S10A and B. *P*-values were obtained by comparing mean PU values for U2AF-repressed and -activated tandem exons with the Wilcoxon–Mann–Whitney test.

DNA mutability generally increases with the length of single- or di-nucleotide runs, leading to diversification over time and shortening of uninterrupted repeats (74,75). In an attempt to capture these evolutionary events in PPTs of U2AF-regulated exon pairs, we aligned their sequences together with their upstream introns, revealing frequent insertions/deletions (indels) in their BP/PPT units (Supplementary Figures S8 and S12). In *U2AF1*, neither the BP cluster of exon Ab nor the ESL had a paralog upstream of exon 3 (Supplementary Figure S8). In *P4HA1* (Supplementary Figure S12), a motif containing a previously mapped BP (59) was upstream of the U2AF-repressed exon 10b but not exon 10a. In *TPM1*, mutations or insertions of adenine-lacking sequences moved the BP of exon 6b upstream. Diversification upstream of *TPM2* exons by indels would shift BP paralogs further upstream of the U2AF-repressed exon 6b, creating a weak distant BP cluster. Indels that remove or create BPs and extend or shorten PPT were observed also upstream of homologous *FYN, ACOX1* and *MAPK14* exons (Supplementary Figure S12).

Collectively, these observations suggest that a lack of U2AF allows the competing ligand(s) (L in Figure 5A) to access longer and more accessible PPTs to activate the first downstream exon and repress its homolog with a shorter PPT. They also suggest that indels involving BP/PPT units played an important role in subfunctionalization of protein isoforms encoded by mutually exclusive exons.

### Tissue-specificity of human *U2AF1* isoforms

Subfunctionalization often involves tissue-specific expression of transcripts carrying either exon of the homologous pair (20,76), however, tissue distribution of human U2AF35 isoforms is not known. We determined the relative abundance of each isoform in 20 tissues, quantified U2AF35 proteins on immunoblots from a panel of cell lines, analyzed RNA-Seq data from 16 human tissues and additional 14 cell lines, and compared their variability in 7 tissues obtained from five rodents (Supplementary Figure S13A–D). The relative abundance of *U2AF1b* was lower than *U2AF1a* in all human tissues examined (Supplementary Figure S13A). The analysis of variance of exon inclusion levels showed that the variability between rodent tissues was significantly higher than variability between strains or species (Supplementary Figure S13B), providing the evidence for minor tissue-specific differences. The lowest expression of both rodent and human *U2AF1b* was found in liver, consistent with the Illumina Body Map RNA-Seq data (Supplementary Figure S13A,B,D). Finally, immunoblotting revealed several heteroploid cell lines, in which U2AF35b was more abundant than U2AF35a (Supplementary Figure S13C).

### Isoform-specific expression of U2AF35 is U2AF65-dependent

**Figure 6.**
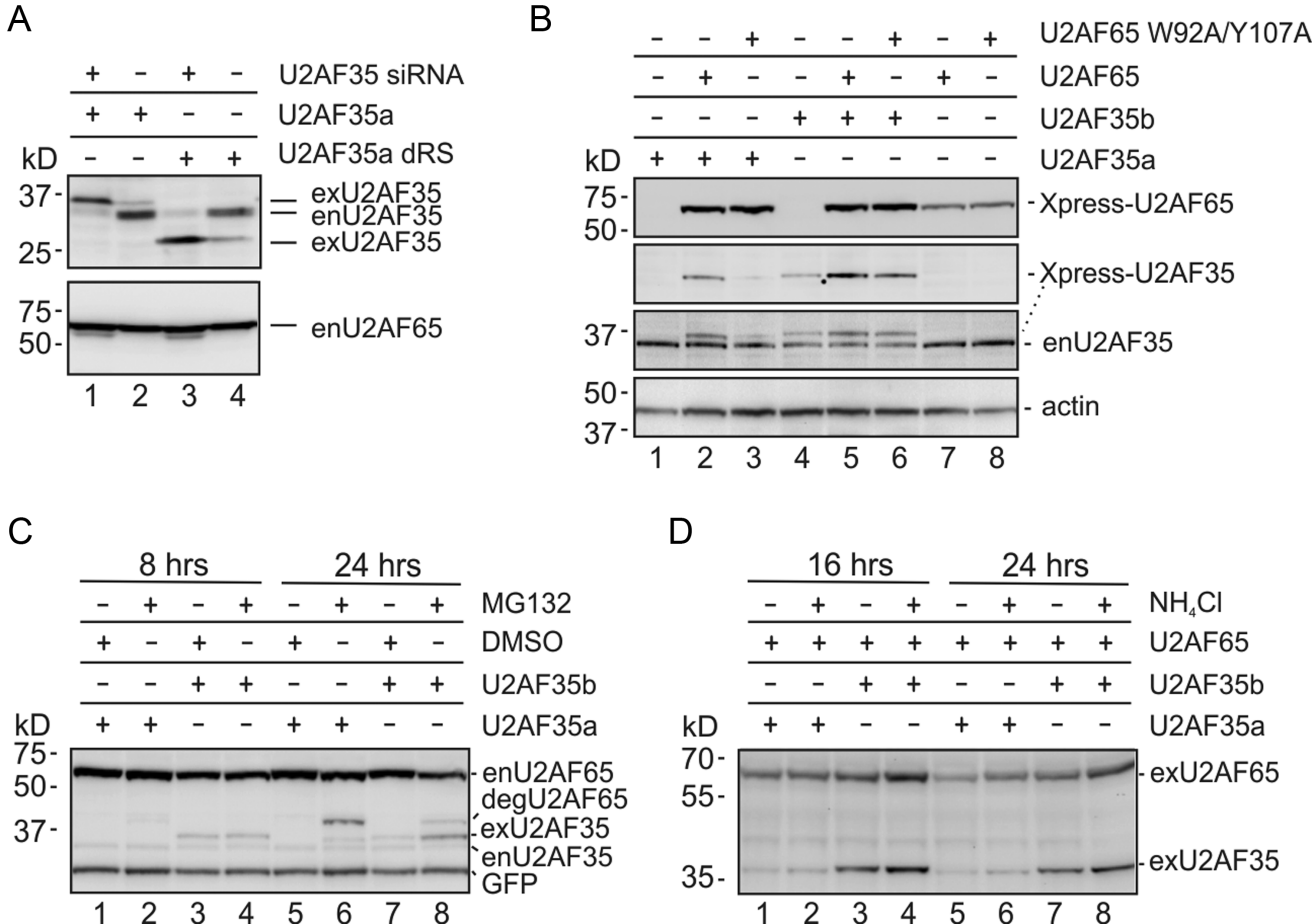
Differential U2AF35a and U2AF35b expression is U2AF65-dependent. (**A**) Increased expression of exogenous U2AF35 by free endogenous U2AF65 (enU2AF65). Concentration of the U2AF35 siRNA and plasmids was 30 nM and 200 ng/ml, respectively. Blots were incubated with antibodies against U2AF35 and U2AF65. Knockdown of U2AF35 was associated with a higher mobility U2AF65 fragment (Supplementary Figure S15). (**B**) U2AF35a and U2AF35b expression depends on their interaction with U2AF65. HEK293 cells were transfected with the indicated Xpress-tagged plasmids and harvested after 48 h. Final concentration of U2AF35 and U2AF65 plasmid DNA in culture media was 130 and 70 ng/ml, respectively. Blots were sequentially exposed to Xpress, U2AF35 and β-actin antibodies. (**C** and **D**) Exogenous U2AF35 isoforms are degraded by the proteasome. Blots were successively incubated with antibodies against Xpress (exU2AF35), U2AF35 (enU2AF35), GFP and U2AF65 (enU2AF65 and degU2AF65) (**C**). A C-terminal degradation product of U2AF65 was described previously in Jurkat cells (78). U2AF expression following addition of lysosomal inhibitor NH_4_Cl and immunoblotting with the Xpress antibody (**D**).

U2AF65 knockdown downregulated U2AF35 (12,77) and increased the U2AF35b/U2AF35a ratio, which was not accompanied by a corresponding increase in the *U2AF1b/U2AF1a* mRNA ratio (12). Following exposure to RNA synthesis inhibitors 5,6-dichloro-1-β-d-ribofuranosylbenzimidazole and actinomycin D, we observed a similar mRNA decay of *U2AF1a* and *U2AF1b* for up to 8 hrs post-treatment (Supplementary Figure S14). Overexpression of exogenous U2AF35 resistant to the U2AF35-specific siRNA (12,19,77) was higher in siRNA-treated than untreated cells (Figure 6A, lanes 1–2), suggesting that free endogenous U2AF65 can enhance exogenous U2AF35 expression. This increase was found also for constructs lacking the U2AF35 RS domain (lanes 3–4). U2AF35 knockdown was associated with the enhanced degradation of U2AF65 (Supplementary Figure S15), possibly through caspase activation (78), which could explain the observed compensatory increase of *U2AF2* mRNAs in depleted cells (12). Expression of U2AF35a and U2AF35b constructs was also increased upon cotransfection with wild-type U2AF65 plasmids into untreated cells (*cf*. lanes 1 versus 2 and 4 versus 5, Figure 6B). Importantly, the U2AF65-mediated enhancement was diminished with U2AF65 constructs mutated in residues that contact U2AF35 (W92, Y107; (22)) as compared to the wild-type U2AF65 (lanes 2 versus 3 and 5 versus 6). The failure of mutated U2AF65 to efficiently augment the signal from U2AF35 proteins was confirmed in independent transfections with increasing amounts of U2AF65 plasmids (Supplementary Figure S16).

The higher expression of U2AF35b than U2AF35a (Figure 6B, lanes 1–3 versus 4–6) (12) could be due to a higher resistance of U2AF35b to degradation, but the U2AF35 degradation pathway is unknown. Overexpression of U2AF35a or U2AF35b in HEK293 cells prior to their exposure to proteasome inhibitor MG132 or lysosomal inhibitor NH_4_Cl (Figure 6C and D) showed an increased signal intensity from both U2AF35a and U2AF35b in cells treated with MG132 for 24 h (Figure 6C, lanes 5, 6 versus 7, 8). Taken together, these results indicate that the isoform-specific expression of U2AF35 is U2AF65-dependent and degradation of each U2AF35 protein is at least partially mediated by the proteasome.

### The role of U2AF35 domains in isoform-dependent expression

**Figure 7.**
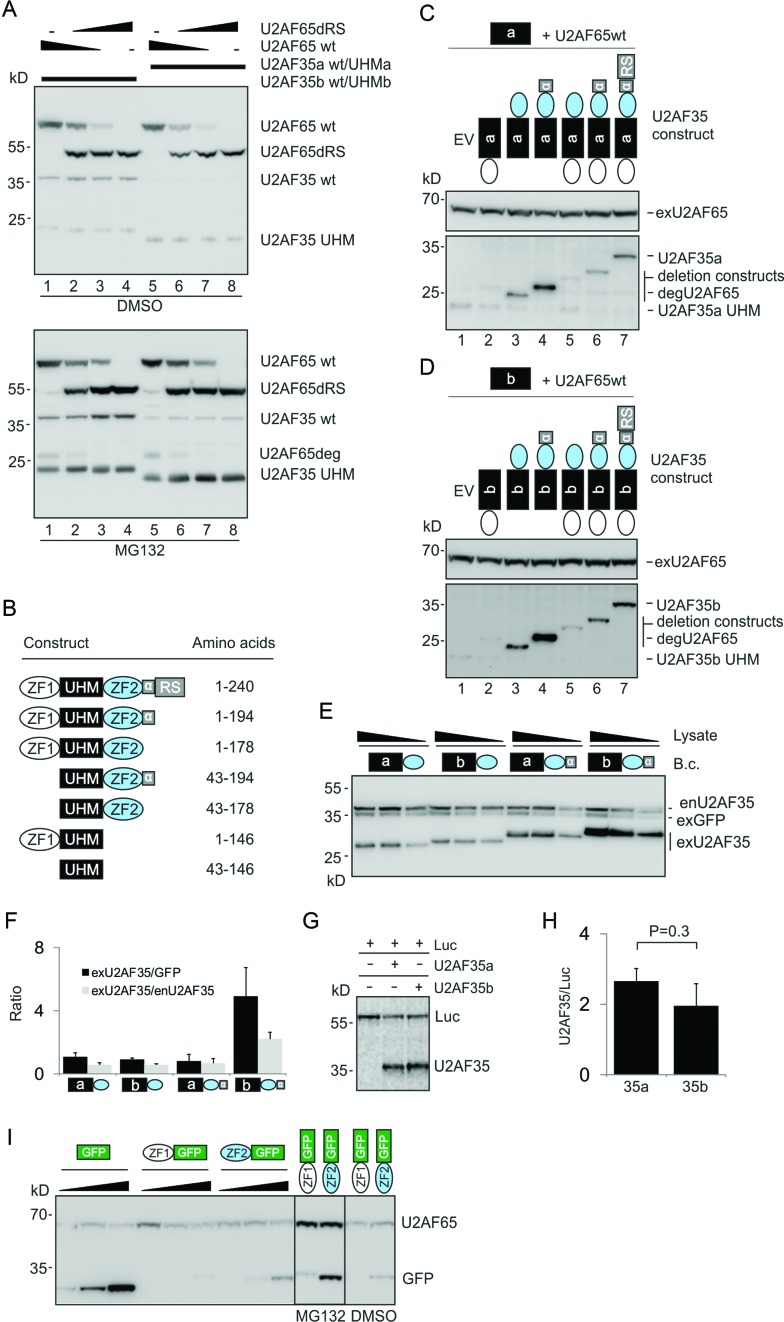
The role of U2AF35 domains in isoform-dependent expression. (**A**) UHM domains alone are not sufficient for differential expression of full-length U2AF35 isoforms. HEK293 cells were transfected with constant amounts of 1:1 mixtures of the wild-type and UHM U2AF35a plasmids or the wild-type and UHM U2AF35b plasmids. The plasmids were supplemented with varying ratios of U2AF65/U2AF65dRS plasmids (150, 50, 15 and 0/0, 100, 135 and 150 ng). The U2AF65ΔRS plasmid was used as a transfection control and to formally exclude that the U2AF65 RS domain can affect the isoform-specific expression through additional contacts with U2AF35. MG132 or DMSO was added 36 hrs later. Immunoblots were incubated with the Xpress antibody, which detects the N-terminal part (∼25 kD) of U2AF65deg (degradation product of U2AF65). U2AF35a was visible only in MG132-treated cells. (**B**) Summary of U2AF35 deletion constructs. Their alignments are in Supplementary Figure S17. The α6 helix (residues 179–194) is denoted by α. (**C** and **D**) The importance of U2AF35 domains for expression of U2AF35a (**C**) and U2AF35b (**D**). The deletion plasmids or empty vectors (EV) were cotransfected with constant amounts of wild-type U2AF65 and UHM-only U2AF35 constructs (150 ng/ml each); membranes were incubated with the Xpress antibody. Plasmid symbols correspond to those in panel (B). (**E**) The α6 helix of U2AF35 is necessary for differential expression of U2AF35a and U2AF35b. Concentration of the indicated bicistronic vectors (B.c.) was 150 ng/ml. Cell lysates (40, 20 and 10 μg for constructs lacking α6 and 30, 15 and 7.5 μg for constructs containing α6) were incubated with U2AF35 and His antibodies. (**F**) Signal intensities measured for panel (E). Error bars indicate SDs. (**G**) Cell-free translation of wild-type U2AF35a and U2AF35b constructs. Luc-expressing plasmid was used as a control. (**H**) Signal intensities for panel (G). Error bars indicate SDs for two independently cloned and sequence-verified plasmids separately translated *in vitro*. (**I**) U2AF35 ZF domains reduce expression of the C-terminal GFP. Expression of the ZF1-GFP constructs could be visualized only after incubation with MG132 (*right panel*). Blots were incubated with the Xpress antibody.

Amino acid differences between U2AF35a and U2AF35b are limited to the UHM (Figure 4A). To determine if this domain alone is sufficient for differential stabilization by U2AF65, we cotransfected plasmids expressing only the U2AF35a or U2AF35b UHM with the corresponding wild-type U2AF35 isoform and varying amounts of U2AF65. Surprisingly, contrary to the full-length constructs, the signal from exogenous UHMa and UHMb was similar (Figure 7A). To identify responsible U2AF35 domains, we cotransfected HEK293 cells with the wild-type U2AF65 expression plasmid and U2AF35a- and U2AF35b-derived, Xpress-tagged deletion constructs (Figure 7B, Supplementary Figure S17). Immunoblotting revealed the highest expression from constructs preserving the α6 helix and lacking ZF1 (Figure 7C and D). In a recent crystal structure of the *S. pombe* ortholog, the α6 helix provides additional contacts with the large subunit and runs in parallel with the α2 helix (23), which differs between vertebrate U2AF35a and U2AF35b (20,22). In contrast to α6, which enhanced the signal from each isoform (lanes 3 versus 4, 5 versus 6, Figure 7C and D), addition of ZF1 diminished their expression (lanes 3 versus 5 and 4 versus 6). To validate these results, we transfected isoform-specific bicistronic constructs expressing GFP and U2AF35 domains into HEK293 cells. Comparison of α6-containing or -lacking plasmids confirmed the higher expression of U2AF35b over U2AF35a in the former constructs, but not in the latter (Figure 7E and F). The same observation was made for their monocistronic counterparts in the presence of increasing concentrations of exogenous U2AF65 (Supplementary Figure S18). In contrast, wild-type U2AF35a and U2AF35b plasmids produced similar protein yields in cell-free reticulocyte lysates (Figure 7B, G and H). Finally, to confirm the effect of individual U2AF35 ZFs on other peptides, we fused each ZF with GFP and transfected the resulting hybrids into HEK293 cells. The GFP signal was diminished by the N-terminally expressed ZF1 while ZF2 reduced the GFP expression to a lesser extent (Figure 7I, Supplementary Figure S19).

In conclusion, the differential expression of U2AF35a and U2AF35b requires interactions between the dimorphic UHM and the α6 helix of U2AF35, most likely through conserved α2/α6 contacts with the U2AF65 ULM. Expression of each U2AF35 protein is also dictated by their ZFs.

## DISCUSSION

U2AF35 isoforms are important for accurate 3′ss recognition (12,20) but their regulation and function in the cell have been obscure. We have first shown that repression of U2AF35b *in vivo* is facilitated by the unusual 3′ss organization of *U2AF1* exon Ab, with weak multiple BPs immediately upstream of its long PPT. Multiple BPs were initially identified for a small number of cellular and viral exons (79–82), but recent RNA-Seq studies suggested that ∼9–32% exons have multiple BPs, up to 11 distinct BPs per exon (57,59). Multiple BPs often cluster in close proximity to each other and are not equally used (59), in agreement with BPs of exon Ab (Figure 2). Exon Ab BPs were not revealed by computational predictions, illustrating their limited accuracy (estimated at ∼75% for the best algorithm (45)). We observed typical A>T substitutions at the 2′-5′ phosphodiester bond (Figure 2D, Supplementary Figure S4C), which are diagnostic of BPs (57,59), but we have not obtained any amplicons indicative of more distant BPs using additional forward primers nearer exon 2 (Figure 2B, Supplementary Figure S4A). BPs of exon Ab and 3 were just upstream of their PPTs (Figure 2A), the arrangement associated with a maximum efficiency of lariat formation (83).

Each BP sequence of exon Ab lacked pyrimidine at position BP-2 (Figure 2A, D), which is the most conserved site flanking the BP adenine (59) and a hot-spot for substitutions leading to genetic disease (84). How are such weak BPs recognized? In yeasts, mutations of BP-2U alter the release of Prp5 from U2, block tri-snRNP association and impair folding of the BP-interacting stem-loop in U2, but do not appear to prevent prespliceosomal formation (85). Apart from auxiliary splicing elements (86), such weak BPs may require enhanced base-pairing with U2, as originally proposed for *GH1* (87). Extending base-pairing contacts between U2 and upstream BP sequences improved splicing of the BP-2U>C mutant (88) and bulged adenosines placed at position +1 or −1 relative to canonical position participated in the first-step splicing catalysis (89). Reversal of the orientation of a base pair switch A_U2_-U_intron_ to U_U2_-A_intron_ resulted in a stacked intrahelical position of the BP adenine and reduced splicing efficiency (90,91), consistent with the importance of nucleophile bulging for splicing (89). The flexibility in nucleophile specification observed in yeast is likely to be even higher for more relaxed mammalian BPs. The number of hydrogen bonds predicted between the weak BPs of exon Ab and U2 snRNA was more than doubled when base-pairing registers of the extended single-stranded regions of U2 were shifted (Figure 2F and G), similar to unusual BPs in *GH1* (87). Thus, future studies of noncanonical BPs should confirm if they can be compensated by shifted or ‘bulged’ registers, as was shown for U1 interactions with the 5′ss (92). Bulged 5′ss were preferentially alternatively spliced, but their fraction estimated at ∼5% (92) is even lower than that of noncanonical BPs (59). Putative shifts in base-pairing interactions between U2 and weak BPs (Figure 2F and G) or between U2 and 5′ss (92) are also reminiscent of translational frameshifting, which alters kinetic partitioning rates between in-frame and out-of-frame codons at ‘slippery’ sites (93).

The weak BPs of exon Ab could be offset by interactions involving unpaired ESL residues (Figure 3). A growing number of hairpins at or near BPs have been reported to affect 3′ss usage (94–101). In *GH1*, the stem-loop stabilities correlated with 3′ss utilization (95), similar to ESL (Figure 3F) and other stem loops (33 and refs. therein). Human introns contain >10 000 of EvoFold-detected structures, with many acting as miRNAs (48), but no miRNA precursors have been described in *U2AF1* (www.mirbase.org). The EvoFold algorithm should thus help identify novel splicing regulatory motifs in many genes, including those containing tandem exons (Table 1). The *U2AF1* ESL could provide a scaffold to support U2 binding to the weak BPs through interactions involving C_−68_/C_−78_ (Figure 3A–C) or promote early BP interactions, as reported for a hairpin that improved binding of the branch-point binding protein (102). It might also act as a kinetic trap for base-pairing shifts, similar to hairpins adjacent to the translational slippery sites (93). ESL–FUBP1/2 interactions (Figure 3D) could prevent misfolding of alternative structures as FUBP1 knockdown and overexpression appeared to differentially affect inclusion of exon Ab in mutant minigenes (Supplementary Figure S7), but interacting residues remain to be defined. Both FUBP proteins were previously implicated in pre-mRNA splicing (103,104). Together with U2AF65, FUBP1 was identified in a multiprotein complex bound upstream of *Tpm2* exon 6b (104), bound a splicing enhancer upstream of *DMD* exon 39 (32) and a cryptic exon in *ATM* (105). Importantly, FUBP1 interacts with PUF60 (106) and SRSF3 (107), which both control exon Ab usage (Figure 1C, D).

The ‘mutually exclusive’ character of *U2AF1* exons Ab and 3 is typical of alternative splicing of duplicated exons, which are present in >10% of human genes (29). Our data suggest that mutation-driven changes in the BP/PPT organization contribute significantly to the evolution of U2AF-regulated tandem exons (Figure 5, Supplementary Figures S8, S10 and S11). Recent RNA-Seq studies showed that deletions upstream of proto-exons favored their creation or maintenance, despite low exonic enhancer densities (108). Longer (>11 nt), U-rich PPTs are preferentially bound by PUF60 (109), but binding preferences of the U2AF-interacting RBM39 (110) remain to be characterized. Each C-terminal UHM in U2AF65, RBP39 and PUF60 (21) can interact with a key U2 snRNP protein SF3B1 (111–115). The U2AF65 UHM can potentially interact with SF3B1 at multiple sites that have distinct binding affinities and mutations of high-affinity sites repressed splicing (112), suggesting that these interactions may compensate weak BPs. Interestingly, cancer-associated *SF3B1* mutations have been recently linked to selection of aberrant upstream BP/PPT units that have shorter PPTs (116).

The PPT signal gradually strengthened in metazoan evolution, with progressive cytosine enrichment from invertebrates to mammals (117), highlighting the importance of cytosine-binding PPT ligands in organisms with high levels of alternative splicing. For example, several fungi lack PPTs altogether and have extended BP consensus while PPTs in zebrafish, which lacks alternatively spliced *U2AF1* exons (Ensembl ENSDARG00000015325), show no cytosine enrichment (117). Longer PPTs in humans have been associated with exon repression by PTBP1 (36) and long U-tracts with major changes in U2AF65 binding upon depletion of hnRNP C (13). Depletion of other candidate exon repressors that bind Y-rich RNAs, including or MBNL1, TIA1 and TIAR, suggested that they may have a more limited and less predictable effect on the U2AF-regulated exon homologs (Supplementary Figure S10C), in agreement with a lack of TIA1/TIAR CLIP tags at U2AF65/hnRNP C binding sites (13). PPT-binding proteins could also contribute to the low exon Ab expression in the liver (Supplementary Figure S13); for example, PUF60 promotes exon Ab (Figure 1D) and is expressed much less in liver than in other tissues (www.proteinatlas.org). Nevertheless, the unpaired character of longer PPTs could also facilitate intramolecular interactions with purine-rich regions, such as exons. Interestingly, differential PU values between activated and repressed homologs were present in the first ∼10-nt of the exon (Supplementary Figure S10C) where U2AF35 bound to a site-labelled pre-mRNA (4).

Younger, primate-specific exons tend to have weaker BPs than established mammalian exons (45) and multiple BPs have been associated with lower evolutionary conservation than single BPs (59). In *FYN*, the U2AF-repressed exon 7a is younger (118) and is preceded by a longer PPT than exon 7b (Table 1, Supplementary Figure S12). In *U2AF1*, both conserved intronic regions are more diverse upstream of exon 3 than exon Ab (Figure 3A), suggesting that the exon with longer PPT also came second. However, the ancestral origin of most U2AF-dependent exon pairs (Table 1) cannot be established at present due to genome assembly uncertainties in arthropods, multiple paralogs and high similarities of duplicated regions (I.V., unpublished data and Peter Gunning, personal communication). In addition, a large fraction of mutually exclusive homologous exons was expressed at very low levels in HEK293 cells or only had one homolog in the mRNA, rendering most cases uninformative. Nevertheless, our results (Figures 1, 2, 5, Supplementary Figure S8 and S12), the association of longer BP-3′ss distances with exon skipping (45,57) and the existence of distant BP outliers (119) indicate that the role of extended PPTs in exon repression and evolution is more important than previously anticipated and further challenge the view that longer vertebrate PPTs always improve exon inclusion over short PPTs.

Our data also show that U2AF-related and SR(-like) proteins are important components of evolutionary processes that assimilated many exon duplication events for the benefit of tissue-specific regulation (Figures 4 and 5, Supplementary Figures S10 and S13). The increase in inclusion of both exons observed for most U2AF-regulated pairs in cells lacking SRSF3 is difficult to explain by chance, as only ∼1% of exons were affected by *Srsf3* knockdown (72), or by a reduced export of two-exon mRNAs for NMD, as both the endo- and exogenous transcripts were more abundant in depleted cells than in controls (Figure 4D and Supplementary Figure S10B). SRSF1-3 interact with U2AF35 *in vivo* (110) and may contribute to the recruitment of U2AF by binding to enhancers (120,121), suggesting that the observed association reflects their physical contacts during spliceosome assembly. The increase of *U2AF1c* at the expense of *U2AF1a* upon SRSF3 knockdown (Figure 4D and E) also supports differential binding to exon Ab/3-containing pre-mRNAs. Among canonical SR proteins, Srsf3 bound to the largest number of substrates, arguing against the ability of other members of the SR family to compensate its loss, despite Srsf3 binding to their NMD switch exons (71,72). A simple ‘BP accessibility model’ where SRSF3 binding to the BP region promotes exon Ab activation does not appear to apply to *Tpm1* (Supplementary Figure S10E). Instead, we propose a model (Figure 5A) in which single-stranded, extended PPTs of repressed homologs attract multiple complexes that compete not only for binding to RNA but also for U2AF-interacting U2 snRNP components, such as SF3B1, SF3A1 or SF3B3 (122). This concept is supported by previous studies showing that SR proteins can promote both exon inclusion and skipping, but their RNA binding patterns or positional effects do not explain such opposite responses (123).

Proper folding of primary transcripts is pivotal to ensure accurate exon recognition from viruses to humans (100,124–126), but the relative importance of RNA folding for splicing decisions is likely to vary in evolution. Long single strands of nucleic acids reassociate orders of magnitude slower than short oligonucleotides (127), and functional long-range intramolecular contacts may be generally less accessible in protein-rich vertebrates than in invertebrates. This may help explain the lack of splicing effects observed for mutations of the longest inverted repeats in the two conserved regions upstream of exons Ab/3, which are highly reminiscent of the selector/docking sites in insects (Supplementary Figure S9). Even if the selector/docking site arrangement is inconsequential in species with a high diversity of proteins involved in structural RNA remodelling such as humans, the reliance of splicing on appropriate local folding has remained critical (Figure 3C, E and F) (51,124–126,128).

Finally, we show that the higher expression of U2AF35b than U2AF35a is U2AF65-dependent and requires interactions between U2AF65 and the α2/α6 helices of U2AF35 (Figures 6, and 7). Dimorphic amino acid positions 59, 61, 65 and 66 in the human UHM (Figure 4A) are in or close to the α2 helix (22,23), yet UHMa and UHMb alone did not recapitulate the differential expression of full-length U2AF35 proteins unless the α6 helix was present (Figure 7B–F). Speculatively, alternative splicing of *U2AF1* could control the orientation of parallel α2/α6 helices in U2AF35 isoforms, provide a means of generating distinct interactions for the negatively charged α2 and affect chaperone activities of U2AF65. The expression of U2AF35 proteins was also differentially affected by ZF1 and ZF2 (Figure 7), confirming that the two ZFs are not equivalent (129), as shown for other proteins with C3H ZFs. For example, ZF1 targeted PIE-1 for degradation in somatic blastomers whereas ZF2 to RNA-rich P granules (130). In the absence of RNA, tristetraprolin ZF1, but not ZF2, adopted a stable fold (131). Together with ZRSR2 and U2AF26, U2AF35 isoforms are unique among proteins with two C3H ZFs in that these ZFs are not strictly in tandem arrangement but are separated by the large UHM, providing an exciting paradigm for future structural studies of these domains and their RNA targets in the context of U2- and U12-dependent splicing.

## ACCESSION NUMBER

E-MTAB-2682.
